# An Advanced Tape-Stripping Approach for High-Efficiency Sampling on Non-Absorbent Surfaces

**DOI:** 10.3390/ijerph191912571

**Published:** 2022-10-01

**Authors:** Pengcheng Zhao, Pak-To Chan, Nan Zhang, Yuguo Li

**Affiliations:** 1 Department of Mechanical Engineering, The University of Hong Kong, Hong Kong SAR 999077, China; 2 Beijing Key Laboratory of Green Built Environment and Energy Efficient Technology, Beijing University of Technology, Beijing 100124, China; 3 School of Public Health, The University of Hong Kong, Hong Kong SAR 999077, China

**Keywords:** film-forming solution, polyvinyl alcohol, qPCR, re-dissolvable membrane, surface microbes, swab

## Abstract

Surface sampling is a frequent task in laboratory work and field studies. Simple methods usually have low efficiency in collecting target substances from surfaces. This study developed an advanced tape-stripping approach for efficient sampling on non-absorbent surfaces. A film-forming solution, prepared using polyvinyl alcohol, is applied to the target surface, where it covers and engulfs the surface deposits and then solidifies into an elastic membrane as it is exposed to air. The deposits are collected by stripping off the membrane and re-dissolving it in water. This new approach exhibited an efficiency of 100% in collecting uniform-size microspheres from glass surfaces and extremely high efficiencies (>96.6%) in detecting selected target DNA materials from glass and stainless steel surfaces. In comparison, the common swab-rinse method exhibited an efficiency of 72.6% under similar measuring conditions. The viability of *S. aureus* during sampling using the new approach decreased as the ethanol concentration in the applied solution increased. Using a solution with a mass ratio of ethanol of 17.6% balanced the effects of multiplication and degradation of the *S. aureus* on glass surfaces during sampling. Overall, the proposed approach exhibits high efficiency in collecting living and abiotic matter from non-absorbent surfaces, complementing existing sampling methods.

## 1. Introduction

Surface sampling is an important step in the detection of surface deposits [1,2]. Sampling efficiency generally refers to the ratio of detected target substances to total target substances each time a surface is sampled. High sampling efficiency and consistency in surface sampling are crucial for research [3,4,5]. However, a sampling efficiency of 100% is difficult to achieve due to losses during the collecting, transporting and detecting processes [6].

**Figure 1:**
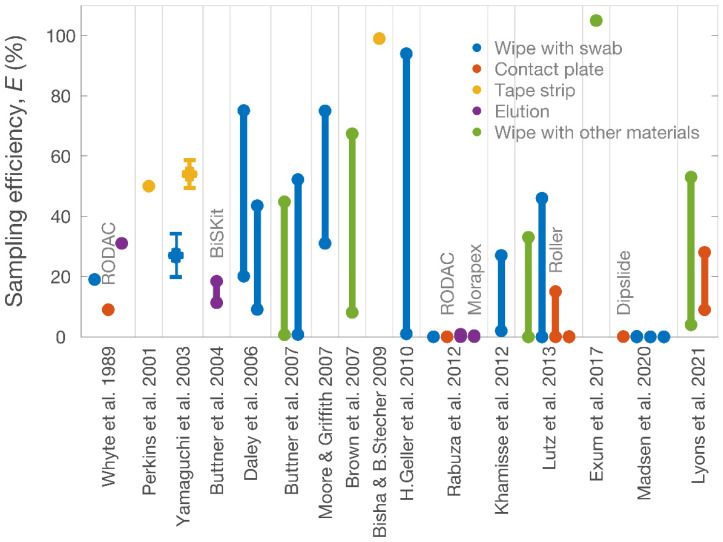
Efficiencies of surface sampling methods evaluated in the 16 studies listed in Table 1. According to the principles of sampling, the studies can be categorized into five groups: wipe with swab (blue), contact plate (red), tape strip (yellow), elution (purple) and wipe with other materials (i.e., sponge, clothing, and gauze; green). Each error bar represents the standard deviation of the data; each vertical line denotes the range of measured values in different measuring conditions. Specific kits or commercial materials used in the samplings are annotated alongside the datapoints.

It is difficult to collect microbes from surfaces because some microbes are likely to undergo irreversible adhesion to substrates [7]. Research on stable and efficient techniques for surface sampling is ongoing [8,9,10]. Table 1 lists recent studies of surface sampling, presenting detailed data on the sampling efficiencies of specific sampling methods. The most widely used sampling methods for collecting surface deposits have efficiencies of <80%, depending on the measuring conditions (see Figure 1). Some commercial sampling kits can achieve higher efficiencies when collecting surface deposits [11,12]. However, efficient sampling techniques usually involve complex operations—it is difficult to simultaneously achieve high sampling efficiency and operational simplicity.

Most surface sampling methods belong to one of the following categories: wipe, contact plate, tape strip and elution [6]. Each method has some limitations: the destructive elution and glove-juice methods are highly efficient in detaching surface deposits but often require complicated post-collection treatments [13]; in contrast, the swab-rinse and contact-plate methods are simple, but their efficiencies are highly variable [14,15,16,17]. Tape-stripping is an efficient approach for collecting surface deposits (Figure 1); however, it suffers from a design conundrum. Strengthening the tape’s adhesiveness increases the collection efficiency but also creates difficulties in detaching the collected material from the adhesive, unless the material on the tape is examined in situ [18,19]. In addition, existing methods can be affected by other factors, such as operational interference [20], surface features [16,21] and the characteristics of the deposits [22].

**Table 1:** Details of 16 studies on sampling methods. Their measured sampling efficiencies are presented in the rightmost column.

Studies	Sampling Targets	Surfaces	Sampling Methods	Sampling Efficiencies (%)
Whyte et al. [23]	Skin bacteria	Skin	Swab	19
			RODAC (contact-plate)	9
			Cylinder (eluting)	31
Perkins et al. [24]	Cytokines	Skin	Sebutape (tape-stripping)	≈50
Yamaguchi et al. [25]	*S. epidermidis*	Plastic	Swab	27 ± 7.2 ^a^
			Adhesive sheet	54 ± 4.6 ^a^
Buttner et al. [12]	*B. atrophaeus* spores	Metal	BiSKit (a foam material)	11.3–18.4 ^b^
Daley et al. [26]	Respiratory epithelial cells	Nasal cavity; nasopharyngeal cavity	Flocked swab	20.1–75.1 ^b^
			Rayon swab	9.1–43.5 ^b^
Buttner et al. [27]	*Erwinia herbicola*	Metal; glass; wood; vinyl; plastic; concrete; nylon	Sponge (+qPCR)	0.7–44.8 ^b^
			Swab (+qPCR)	0.8–52.2 ^b^
Moore and Griffith [28]	*Escherichia coli*; *S. aureus*	SS	Swab	31–75 ^b^
Brown et al. [29]	*B. atrophaeus* spores	SS; painted wallboard	Wipe (by polyester-rayon blend gauze)	8.1–67.4 ^b^
Bisha and Brehm-Stecher [30]	*Salmonella*	Tomato	Adhesive tape	>99
Hong-Geller et al. [22]	*B. anthracis*; *Yersinia pestis*	Plastic; SS; glass; vinyl	Swab/wipe	<1–94 ^b^
Rabuza et al. [11]	*S. aureus*; *Klebsiella pneumoniae*	Fabric pieces	Swab	<0.01
			RODAC (contact-plate)	<0.01
			Destructive elution	0.07–0.85 ^b^
			Morapex device	0.10–0.36 ^b^
Khamisse et al. [31]	Bacterial DNA	Polyvinyl chloride; SS	Swab	2–27 ^b^
Lutz et al. [32]	*S. aureus*	SS	Electrostatic wipe	0–33 ^b^
			Swab	0–46 ^b^
			Roller sampler	0.01–15 ^b^
			Contact-plate	0–0.09 ^b^
Exum et al. [15]	*Escherichia coli*	Surfaces in households	Dry cloth	105
Madsen et al. [9]	*S. aureus*	SS	Dipslide	0.08–0.10 ^b^
			eSwab	0–0.16 ^b^
			Viscose swab	0–0.02 ^b^
			Cotton swab	0–0.02 ^b^
Lyons et al. [10]	*Clostridioides difficile* spores; *Klebsiella pneumoniae*; *S. aureus*; *Acinetobacter baumannii*	Nitrile glove	Wipe (by cellulose sponge)	4–53 ^b^
			Contact-plate	9–28 ^b^

Abbreviations: RODAC—replicate organism detection and counting; *S.*—*Staphylococcus*; *B.*—*Bacillus*; SS—stainless steel. ^a^ Mean ± standard deviation. ^b^ The range represents the measured values in different measuring conditions.

In this study, a novel approach for surface sampling is proposed. A polyvinyl alcohol (PVA) solution was prepared and applied to the target surface. The solution collects surface deposits as it solidifies into an elastic membrane that can be stripped off. The membrane releases the collected material when it is re-dissolved in water. The efficiencies of this new approach in sampling DNA materials and uniform-size microspheres from different surfaces were measured. In addition, the effect of the PVA solution on bacterial viability was tested. Although the proposed method, an advanced tape-stripping technique, is not suitable in all situations, it provides an option with high sampling efficiency and low cost that can complement existing methods for surface sampling.

## 2. Materials and Methods

### 2.1. Literature Review of Surface Sampling Methods

Sixteen articles on surface sampling, published in the 1989–2021 period, were retrieved by searching for the keywords “surface sampling”, “sampling efficiency”, “swab”, “contact plate”, “tape strip”, “elute” and “wipe”. Another criterion for selection was that the article should describe a systematic study on one or more sampling methods and clearly report the corresponding sampling efficiencies. It was difficult to retrieve all studies that met these criteria. Nevertheless, the chosen articles were not deliberately selected (e.g., articles reporting low sampling efficiencies) to emphasize the good qualities of the high sampling efficiency of the approach proposed in this study.

### 2.2. Materials

Four reference strains with double-stranded DNA as genetic materials, comprising a Gram-positive bacterium, *Staphylococcus aureus* ATCC 25923, a Gram-negative bacterium, *Escherichia coli* ATCC 25922, a fungus, *Saccharomyces cerevisiae* ATCC 9763, a bacteriophage, P22 ATCC 97540, and a type of fluorescence microsphere were applied to different surfaces. *Salmonella enterica serovar* Typhimurium ATCC 14028 was incubated to be the host for culturing phage P22. Lyophilized powders of *S. aureus*, *E. coli*, *S. cerevisiae* and *S. Typhimurium* were purchased from HKM, Guangdong, China. The bacteriophage P22 stock was purchased from Laval University, Canada. Fluoro-Max Green Particulate Marker Particles, fluorescence microspheres sold in powder form (catalogue number: 35–3; material: polystyrene–divinylbenzene; average diameter = 8 μm; coefficient of variation < 18%) were purchased from ThermoFisher, Hong Kong.

Two types of flat surfaces were prepared. Glass surfaces were obtained by directly using typical glass petri dishes with an average surface roughness (*Ra*) of ≈ 0.01 μm, measured using a surface roughness tester (TR200, Beijing Kaida Technology Development Co., Ltd., Beijing, China). Stainless steel surfaces were obtained by cutting stainless steel 301 plate (with a thickness of 1 mm and a measured *Ra* of ≈ 1 μm) into 10 cm × 10 cm coupons.

Film-forming solutions were prepared with four ingredients: (I) PVA powder (#1788; degree of polymerization = 1700; degree of alcoholysis = 88%), acquired from Shandong Usolf Chemical Technology Co., Ltd., linyi, China; (II) ethanol absolute for analysis (purity ≥ 99.5%; CAS: 64-17-5), acquired from Anaqua Global International Inc., Ltd., Hong Kong; (III) glycerol aqueous solution for analysis (86–88% wt%; CAS: 56-81-5), acquired from Acros Organics; and (IV) nuclease-free water for molecular biology (prepared from double distilled and deionized water; CAS: 7732-18-5), acquired from Sigma-Aldrich, Hong Kong.

The glycerol solution was also mixed with appropriated mass of nuclease-free water to obtain “50% glycerol” (i.e., glycerol aqueous solution; ≈50% wt%) for aliquot storage. Flocked swabs, FLOQSwabs^®^ 552C, COPAN, were used to sample the surfaces using the swab-rinse method and used as an auxiliary tool in the proposed approach. Lysogeny broth (LB) and agar powders were purchased from Hangzhou BAISI Biotechnology Co., Ltd., Hangzhou, China. Powders of yeast extract peptone dextrose (YPD) medium were obtained from Shanghai RUICHU BioTech Co., Ltd., Shanghai, China.

### 2.3. Surface Preparation

#### 2.3.1. Inoculum Preparation

Four reference strains were prepared following the protocol described in our previous study [33]. Each bacterial lyophilized powder was mixed with 1 mL of the reviving broth (i.e., a brain heart infusion broth) provided in the product package, and transferred into 5 mL LB for overnight culture at 37 °C. Frozen aliquots were obtained by mixing 5 mL of the culture broth with 5 mL of 50% glycerol and aliquoting 500 μL into each tube for storage at −80 °C. The *S. cerevisiae* lyophilized powder was revived using a similar process; however, the reviving broth was not used and the culture was kept in YPD medium at 25 °C. P22 stock was directly mixed with an equal volume of 50% glycerol, and 500 μL aliquots were transferred into each tube for storage at −80 °C.

Frozen aliquots of *S. aureus*, *E. coli* and *S. Typhimurium* were transferred into separate flasks containing 25 mL of LB, incubated overnight at 37 °C on an orbital shaker (130 rpm) and streaked for isolation on agar plates (LB with 1.5% agar). Then, a colony of each bacterium was sub-cultured in a flask with 25 mL LB and incubated for 15 h at 37 °C in an orbital shaker (130 rpm). Frozen aliquots of *S. cerevisiae* were transferred into a flask containing 25 mL YPD medium and incubated for 8 h at 25 °C in an orbital shaker (130 rpm). A 10 μL P22 stock sample was added to 25 mL of the prepared *S. Typhimurium* suspension; the newly mixed suspension was cultured for 24 h at 37 °C.

Suspensions of *S. aureus*, *E. coli* and *S. cerevisiae* were diluted to a concentration of 10^4^–10^5^ CFU/μL. Phage P22 was isolated from the host by centrifuging at 5000 rpm for 15 min, obtaining a supernatant in which the phages had a concentration of 10^7^–10^8^ PFU/μL, quantified by plaque assay. The prepared suspensions of each strain were aliquoted into separate tubes at 4 °C for less than 24 h and vortexed for 5 s before use.

#### 2.3.2. Surface Seeding

The glass and stainless steel materials were autoclaved at 121 °C for 20 min and air-dried in a biosafety cabinet overnight before use. For each surface, a circular area with a diameter of 5 cm was defined as the target area for microbial inoculation and sampling. For each glass petri dish, the target area was marked on the outer side of the dish using a marker pen; for each stainless steel coupon, the sampling area was estimated with the naked eye. For each type of reference strain, a 200 μL microbial suspension was dispensed within the target area on a prepared surface (a stainless steel coupon or the inner side of a glass petri dish) and air-dried to visible dryness. For the microspheres, approximately 5 mg of microsphere powder was directly spread within the target area on a glass surface.

### 2.4. Surface Sampling

#### 2.4.1. Preparation of PVA Solutions

A liquid solution for surface sampling was prepared as follows. First, 2.5 g of PVA powder was dissolved in 50 g of nuclease-free water by stirring at 95 °C for 60 min; 1 g of glycerol was added, and then the mixture was autoclaved at 121 °C for 20 min; after it cooled to room temperature, the sterilized mixture was further mixed evenly with 25 g of ethanol absolute [34,35]. Thus, a PVA solution was obtained, with a mass ratio of ethanol in the entire solution (*m_e_*) of 32%, which is considered as the default mass ratio of ethanol in the following discussion unless *m_e_* is otherwise specified. PVA solutions with a series of other values of *m_e_*, i.e., 0, 3%, 6%, 10%, 18% and 25%, were prepared by adjusting the masses of water and ethanol during preparation.

#### 2.4.2. Surface Sampling Using PVA Solution

**Figure 2:**
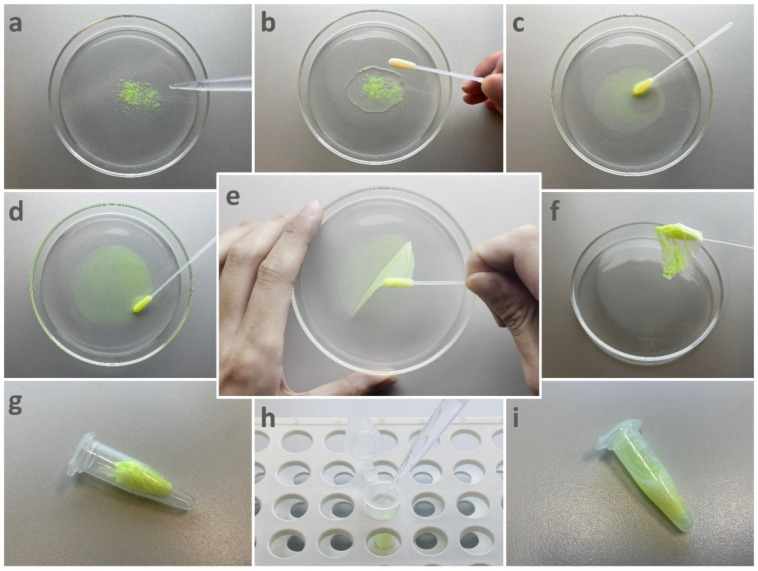
Demonstration of the new sampling approach. Fluorescence microspheres were sampled from a glass plate using the PVA solution. The sampling process is illustrated step-by-step as follows: (**a**) the PVA solution is dispensed into a contaminated glass surface; (**b**) a swab is used to wipe the glass surface; (**c**) the swab tip remains in contact with the solution, as the solution air-dries; (**d**) the solution solidifies into an elastic membrane; (**e**) the attached swab is lifted; (**f**) the membrane is stripped off; (**g**) the swab tip and the attached membrane are moved to a tube; (**h**) water is added into the tube; (**i**) the membrane is re-dissolved, releasing the sample. A short video demonstrating the sampling process is provided in Video S1.

The new surface sampling approach is illustrated step-by-step in Figure 2. First, 1 mL of the prepared PVA solution was dispensed onto the target area (Figure 2a,b), and was then spread evenly using a sterile dry swab to cover the surface deposits in the target area (Figure 2c). The swab was used to continually wipe the target area for approximately 30 s to facilitate the mixing of the surface deposits with the PVA solution (Figure 2c). The PVA solution was allowed to air-dry and solidify into an elastic membrane, which required 1–2 h under the laboratory conditions. During this drying process, the swab tip was kept static and in contact with the drying solution (Figure 2d). Then, the elastic membrane was stripped from the surface by pulling on the swab tip (Figure 2e,f). The PVA membrane along with the swab tip was cut off using sterile pliers and moved into a tube (Figure 2g). Finally, 1 mL of nuclease-free water was added to the tube, and the tube was vortexed for 60 s (Figure 2h). The collected materials were released and resuspended as the solidified membrane re-dissolved in water (Figure 2i).

The PVA solution applied on surfaces had an evaporating speed of approximately 10 mg/min under the laboratory measuring conditions. In the laboratory, air temperature was maintained at 22 ± 1 °C, relative humidity was maintained between 65% and 75%, and a slightly greater than normal air flow was generated from air-conditioning. The evaporating speed was slower for PVA solutions containing less ethanol.

#### 2.4.3. Surface Sampling Using Swab

The common swab-rinse technique was also used to sample the surface. A sterile swab rinsed with 1 mol/L of phosphate-buffered saline solution was used to sample a surface by repeatedly wiping the target area over a 10 s period; then, the swab tip was cut off and moved into a tube. Then, 1 mL of nuclease-free water was added to the tube, and the tube was vortexed for 60 s.

### 2.5. Measurement of Sampling Efficiency

Sampling efficiency (*E*) is defined as the ratio of the quantity of target substances detected on a surface at a specific time of sampling (Δ*C*) to the quantity of target substances on the site just before their collection (*C*) (Equation (1)). For the new sampling approach, the collection was defined to occur at the moment at which the formed membrane was stripped off.

(1) $$E=\frac{\Delta C}{C}\times 100\%$$

In general, if the real *E* is very close to 100%, measurement errors can result in an estimated *E* exceeding 100% [15,22]. Thus, the *E* value is measured using the following sequential-sampling method [23,36]: a given surface is sampled *N* times, and the *E* is calculated using the quantities collected in the *N* samples, i.e., Δ*C*_1_, Δ*C*_2_, …, Δ*C_N_*. According to Equation (1), after the first sampling, the quantity of sampling target substances remaining on the surface is

$$C-\Delta C_{1}=C-EC=\left(1-E\right)C$$

Assuming that (I) the sampling efficiency remains constant and (II) the sampling target substance does not multiply or degrade during the consecutive *N* samplings, it can be inferred that after the second sampling, the quantity of sampling target substances remaining on the surface is

$$C-\Delta C_{1}-\Delta C_{2}=C-EC-E\left(C-\Delta C_{1}\right)={\left(1-E\right)}^{2}C$$

As such, after the *n*-th sampling, the quantity of sampling target substances remaining on the surface is

$$C-\sum_{i=1}^{n}\Delta C_{i}={\left(1-E\right)}^{n}C$$

Then, Equation (2) is derived from the above relationship.

(2) $$\sum_{i=1}^{n}\Delta C_{i}=\left[1-{\left(1-E\right)}^{n}\right]C$$

According to Equation (2), the quantity of sampling target substances in the *n*-th sampling (Δ*C_n_*, 1 ≤ *n* ≤ *N*) can be derived as in Equation (3).

(3) $$\Delta C_{n}=\sum_{i=1}^{n}\Delta C_{i}-\sum_{i=1}^{n-1}\Delta C_{i}=\left[E{\left(1-E\right)}^{n-1}\right]C$$

Then, a relationship between Δ*C_n_* and *n* is determined by combining Equations (2) and (3), as in Equation (4).

(4) $$\frac{\Delta C_{n}}{\sum_{i=1}^{N}\Delta C_{i}}=\frac{E{\left(1-E\right)}^{n-1}}{1-{\left(1-E\right)}^{N}}$$

where, *E*, as the only unknown parameter, can be evaluated by fitting a series of measured data (*n*, Δ*C_n_*/$\sum_{i=1}^{N}$Δ*C_i_*) with Equation (4). Thus, *E* can be calculated using the quantity of sampling target substances detected in the *N* samples (i.e., Δ*C*_1_, Δ*C*_2_, …, Δ*C_N_*) based on this assumed relationship.

After the *N* sequential sampling procedures, one extra sampling procedure on the same site was performed using the swab-rinse method to validate the effectiveness of the new sampling approach—if the amount of sampling target substances on the final swab was not significantly more than the amount in each of the former samples, this would suggest that (I) almost all of the target substances had been removed from the surface and (II) the sampling target substances collected in the *N* samples did not significantly degrade.

### 2.6. Experimental Design

Fifteen sets of experiments were performed under different conditions, as listed in Table 2. The sets of experiments were categorized by two objectives: (I) to measure *E* under different sampling conditions; and (II) to measure *S. aureus* viability in the PVA solutions with different *m_e_*. Before each set of tests, a negative-control test was performed by sampling a clean surface using the PVA solution to ensure that the prepared solution was free of the sampling target substances. Subsequent tests were valid only if the quantity of sampling target substances in the prepared solution in this validation test was below the detection limit. For each set of experiments, the results of 5 or 6 replicates were averaged (see Table 2).

#### 2.6.1. Sampling Efficiencies under Different Conditions (Sets 1–8)

In sets 1–7 (Table 2), specific short DNA fragments were selected (listed in Table 3) for measuring the sampling efficiency in sampling the DNA of different microbes, as short DNA fragments are relatively stable in the environment and do not replicate in the PVA solution (*m_e_* = 32%) [37]. The DNA concentration in each sample was quantified by carrying out DNA extraction, followed by qPCR [33]. A 600 µL sample from the collecting tube was transferred to the PowerBead tube provided in the DNA isolation kit (DNeasy PowerSoil Kit, Qiagen) for DNA extraction. Before DNA extraction, 100 µL of phenol–chloroform–isoamyl alcohol solution (25:24:1, *v*/*v*) was added to the PowerBead tube to promote the removal of proteins from nucleic acids. The purified DNA was then quantified using a qPCR detection system (CFX Connect, Bio-Rad). iTaq Universal SYBR Green Supermix (CFX Connect, Bio-Rad) was used as the reagent for the 10 μL reaction volume. The DNA fragment and the cycling conditions in qPCR assay for each type of microorganisms are listed in Table 3. Calibration of the qPCR amplification efficiency for each pair of primers is provided in Supplementary File S2.

**Table 3:** The source, length and primers of the chosen DNA fragment and the cycling conditions in qPCR for each type of microorganism.

*S. aureus* and *E. coli* [38]	
DNA fragment	16s ribosomal DNA (rDNA)
Number of base pairs	178 bp
Primers (5′–3′)	(*341-F*) CCT ACG GGA GGC AGC AG (*518-R*) GTA TTA CCG CGG CTG CTG
Cycling conditions	95 °C × 1 min + [95 °C × 10 s + 60 °C × 30 s] × 40 cycles
Amplification efficiency	95.3%
*S. cerevisiae* [39,40]	
DNA fragment	Internal Transcribed Spacer-2 (between 5.8S rDNA and 28S rDNA)
Number of base pairs	≈350 bp
Primers (5′–3′)	(*ITS3-F*) GCA TCG ATG AAG AAC GCA GC (*ITS4-R*) TCC TCC GCT TAT TGA TAT GC
Cycling conditions	95 °C × 1 min + [95 °C × 30 s + 60 °C × 30 s + 72 °C × 1 min] × 40 cycles
Amplification efficiency	82.6%
P22 [41]	
DNA fragment	A segment from the 14,567th to the 14,617th base pair in the complete genome sequence
Number of base pairs	51 bp
Primers (5′–3′)	(*14567-F*) CTT AAC AAG CTC TGA CTG CTC ATC A (*14617-R*) CCA TCG CCT GTG ACT GGA T
Cycling conditions	95 °C × 1 min + [95 °C × 10 s + 60 °C × 30 s] × 40 cycles
Amplification efficiency	97.7%

After subjecting a sample to qPCR, a *C_q_* value for the target DNA fragment was obtained. According to the working principle of qPCR, the DNA concentration in a sample (Δ*C*) is proportional to a function of *C_q_* (Equation (5)).

(5) $$\Delta C\propto {\left(1+e\right)}^{-C_{q}}$$

where *e* is the amplification efficiency in the qPCR process, and the value for each piece of DNA fragment is calibrated (see Supplementary File S2). Thus, the left-hand side of Equation (4) can be calculated from the raw qPCR data, as derived in Equation (6).

(6) $$\begin{array}{ll} \frac{\Delta C_{n}}{\sum_{i=1}^{N}\Delta C_{i}} & =\frac{\Delta C_{n}}{\Delta C_{1}+\Delta C_{2}+\cdots +\Delta C_{N}}\\ & =\frac{{\left(1+e\right)}^{-C_{q\left(n\right)}}}{{\left(1+e\right)}^{-C_{q\left(1\right)}}\;\;\;+{\left(1+e\right)}^{-C_{q\left(2\right)}}\;\;\;+\cdots +{\left(1+e\right)}^{-C_{q\left(N\right)}}}\\ & =\frac{1}{\sum_{i=1}^{N}{\left(1+e\right)}^{\left[C_{q\left(n\right)}\;\;\;-C_{q\left(i\right)}\;\;\right]}} \end{array}$$

**Figure 3:**
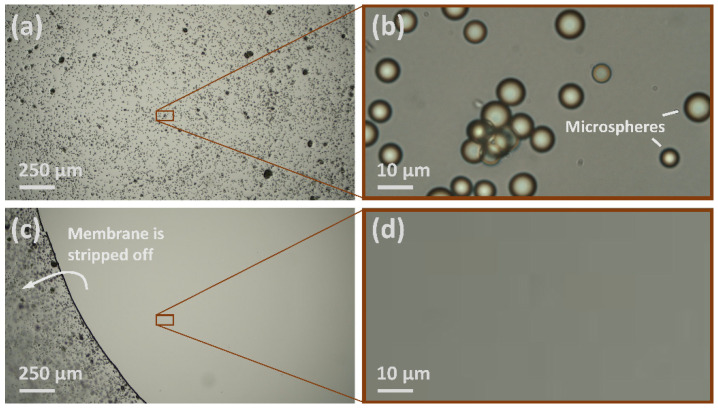
Digital microscope images demonstrating the process of sampling surface microspheres using the proposed approach. (**a**) Microspheres on a glass surface covered by the PVA solution, air-dried to a solid membrane; (**b**) a magnified area of 100 μm × 56 μm; (**c**) removal of the surface microspheres by stripping off the membrane; (**d**) the magnified area after stripping off the membrane.

In set 8 (Table 2), the number of surface microspheres was quantified by directly counting the surface microspheres using a digital microscope (i.e., an optical microscope connected to a high-resolution camera and a monitor). A glass petri dish was fixed on the mechanical stage of the microscope. The microspheres were dispensed to the inner side of a glass petri dish and sampled multiple times on-site. A rectangular area of ≈100 μm × 56 μm on which microspheres were deposited was magnified and displayed on the monitor (Figure 3b,d). The number of microspheres removed from this area in one sampling process (Δ*C_n_*) is the difference in the number of microspheres before and after the sampling, as shown in Figure 3b,d.

#### 2.6.2. *S. aureus* Viability in the PVA Solutions (Sets 9–15)

In the laboratory environment, the 1 mL of PVA solution on a 5-cm diameter circular area took 1–2 h to dry completely, during which the microbes engulfed in the solution could multiply or degrade. Thus, the number of microbial cells present at the moment the membrane is stripped off (*C*) is different from the number of microbial cells present when the PVA solution is applied to the surface (*C*_0_), with a multiplication rate of *R* = *C*/*C*_0_.

In the case of *S. aureus* on glass surfaces, it is possible to determine an optimal mass ratio of ethanol in the PVA solution to achieve *R* = 1. A 200 μL *S. aureus* suspension was inoculated on the inner side of a glass petri dish (i.e., a glass surface), air-dried and coated with the PVA solution with a specific *m_e_* (sets 9–15 in Table 2). After the PVA solution solidified into a membrane, 20 mL of nuclease-free water was dispensed into the glass petri dish to re-dissolve the membrane on-site. Then, 1 mL of this solution was taken to perform plate counting to estimate the number of living *S. aureus* cells on the surface at the moment the PVA solution was completely solidified, thus obtaining the value of *C*.

Simultaneously, *S. aureus* (from the same tube of *S. aureus* used in the above trial) inoculated on a glass surface was air-dried and directly mixed on-site with 20 mL of nuclease-free water. Then, 1 mL of this solution was taken to perform plate counting to estimate the number of living cells of *S. aureus* on the surface before sampling, obtaining the value of *C*_0_.

Then, the multiplication rate for the *S. aureus* was calculated as *R* = *C*/*C*_0_, with *R* > 1 (or < 1) meaning that the surface *S. aureus* multiplied (or degraded) in the PVA solution during its solidification, and *R* = 1 means that the effects of multiplication and degradation counteracted each other.

## 3. Results

### 3.1. Sampling Efficiency of the New Approach

**Figure 4:**
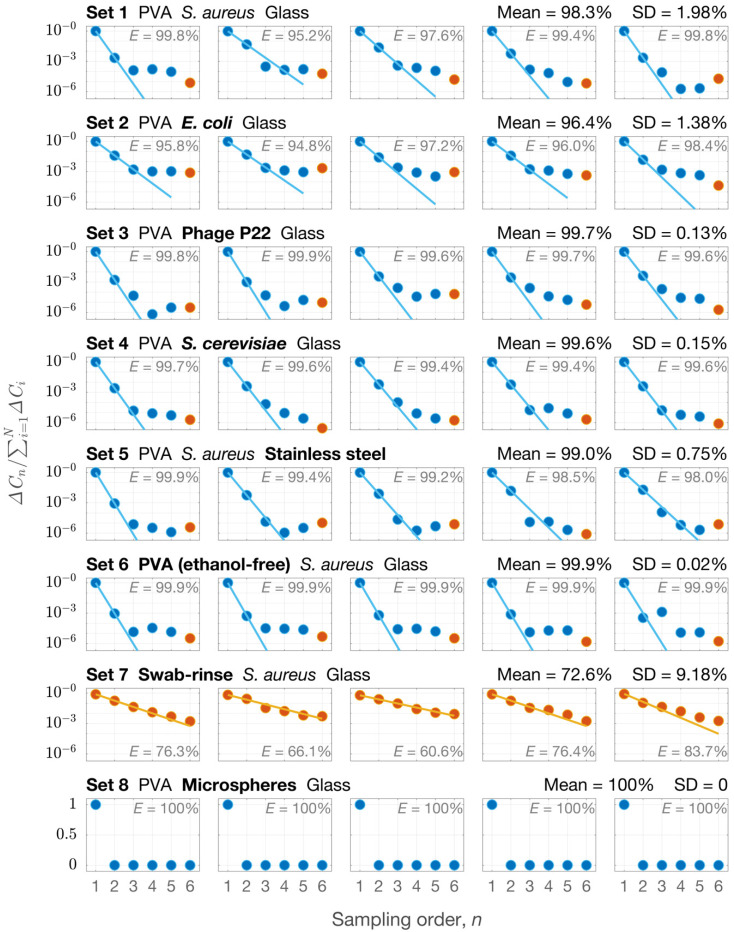
Evaluation of the sampling efficiencies (*E*) for the new approach (sets 1–6 and 8) and the swab-rinse method (set 7). *E* was evaluated through *N*-times sequential sampling, denoted by circles in a plot (blue: new approach; orange: swab-rinse method). Sampling efficiency (*E*) was evaluated by fitting the datapoints with Equation (4) and is shown as lines. In each set, the mean and SD of *E* were obtained from five *E* measurements.

The efficiencies of the new approach in sampling DNA materials on surfaces were >96% under all the measuring conditions (sets 1–6 in Figure 4), with an average of 98.8%. After five sequential sampling processes on a surface, minute DNA concentrations could be detected by the swab-rinse method, indicating that the new sampling approach effectively collected microbes from both types of surfaces, and the DNA in the PVA solutions exhibited no significant degradation.

As shown in Figure 4, most of the *E* values measured by the sequential sampling method were greater than 99%. Sampling *E. coli* on glass surfaces (set 2 in Figure 4) was slightly less efficient, but still exhibited an average *E* of 96.4%. When using ethanol-free PVA solution to sample *S. aureus* on glass surfaces (set 6 in Figure 4), the average *E* of 99.9% might be overestimated, because living *S. aureus* can multiply in an ethanol-free PVA solution. The relationship between the *S. aureus* multiplication and the overestimation of the *E* value is derived in Supplementary File S2. Nevertheless, the decreasing trend of the datapoints in each plot in sets 1–6 (Figure 4) indicates an extremely high efficiency of the new technique in detaching target substances from the surfaces.

In Figure 4, the fourth and fifth points in some plots deviated slightly upward from the fitting lines, and this deviation appears to be amplified on the logarithmic-scale *y*-axes. This could be due to errors in DNA quantification when there are excessively low concentrations of the target DNA fragments. For example, false positives may occur in qPCR tests due to the incorrect annealing of primers [42].

For comparison, the swab-rinse method was used to consecutively sample a surface (set 7 in Figure 4), obtaining an average *E* of 72.6% with a standard deviation (SD) of 9.18%. Both the sampling efficiency and the repeatability of the conventional swab-rinse method in the measuring condition were worse than those of the new approach.

Glass surfaces with uniform-size microspheres were sampled using the PVA solution (set 8 in Figure 4). A 100% sampling efficiency was obtained, as in each set of sequential sampling, all of the microspheres in the magnified area examined (as illustrated in Figure 3) were removed from the surface in the first sampling.

### 3.2. S. aureus Viability in the PVA Solutions

**Figure 5:**
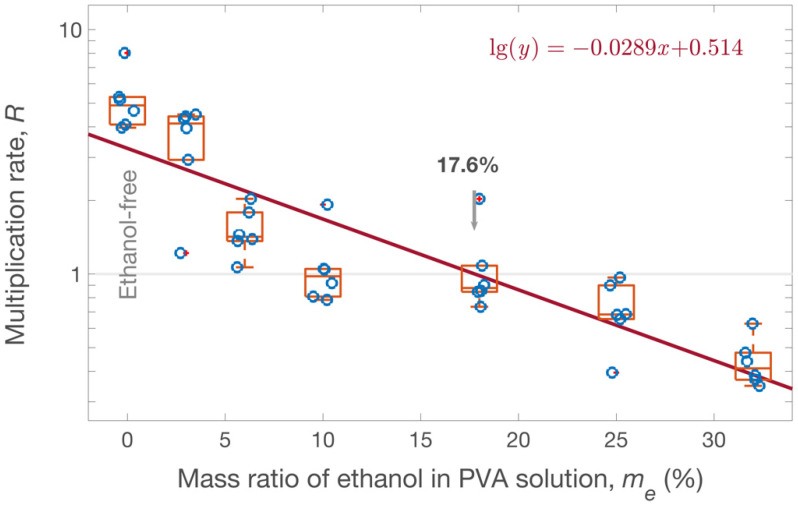
Effect of PVA solutions on *S. aureus* viability. *S. aureus* multiplication rate (*R*) in the PVA solutions with different *m_e_* was evaluated. Each *R* value, the average of six replicates, is illustrated in the boxplot along with a jittering scatter plot. An *m_e_* of 17.6% was obtained by data-fitting (red line) to balance the effects of multiplication and inhibition of *S. aureus*.

The effects of the PVA solutions with different *m_e_* values on *S. aureus* viability are shown in Figure 5. A PVA solution of *m_e_* = 32% inactivated 30–60% of *S. aureus*; in contrast, the ethanol-free PVA solution (*m_e_* = 0) provided a favorable aquatic environment for bacteria, so the *S. aureus* multiplied to reach 3–5 times the number in the original inoculation. According to the data fitting (red line in Figure 5), the PVA solution with an *m_e_* ≈ 17.6% led to a constant concentration of *S. aureus* (i.e., *R* = 1). In that sample, the inhibition of microbial growth due to ethanol and its promotion by the aquatic environment counteracted each other.

It is known that the multiplication of *S. aureus* can lead to an increase in DNA concentration when the inactivation of *S. aureus* is faster than the degradation of DNA. Thus, a PVA solution with an *m_e_* > 17.6% can be used to sample DNA materials. In practice, to maintain a constant quantity of sampled organic or living substances during the sampling process, the optimum ethanol concentration in the PVA solution should be varied according to the target materials (e.g., different microbial species and genetic materials).

## 4. Discussion

### 4.1. Significance of This Study

In this study, we proposed a new approach to surface sampling using a film-forming agent, i.e., PVA solution. This solution, with low toxicity, high water solubility and chemical resistance, is suitable for collecting a variety of substances from surfaces [43]. The PVA solution can be considered a “soluble tape”, as it can solidify to an elastic membrane when exposed to air, which can in turn be stripped off and re-dissolved in water. The cost of all of the ingredients in the prepared solution per milliliter is negligible in comparison with the cost of each swab used in this study.

The new approach offers a high and stable efficiency for collecting target substances from surfaces, which is independent of the surface area, the stripping movements, the existence of impurities and the selection of the auxiliary tools (e.g., sterile swabs and forceps). It minimizes the errors introduced by operational interference, such as wetting the swab head with appropriate buffer, squeezing the excessive buffer and swabbing surfaces in the swab-rinse technique. Compared with normal tape-stripping methods, applying the PVA solution to surfaces can efficiently collect multiple layers of deposits and completely release the collected materials in water. Despite some limitations, the new approach complements existing sampling methods and could be developed as a sampling tool for specific jobs such as collecting trace substances from ancient remains [44], cleaning instrument surfaces [45] and collecting DNA at crime scenes [46].

### 4.2. Sampling Mechanism

Existing sampling methods (see Table 1) can be categorized as “wipe”, “contact”, “adhere” or “elute” types. Microorganisms on a surface can form strong adhesions [47], which can be overcome by sampling tools, such as rinsed swabs and elution buffers. However, in conventional sampling methods, the “collection” of surface deposits occurs in a two-dimensional interface—the sampling tool and the surface are competing to adsorb the deposits. In contrast, in the new approach, the PVA solution engulfs surface deposits, as it is applied to a surface. In this scenario, most of the deposits, comprising both sampling target substances and other impurities, will already be detached from the surfaces before the solution solidifies to a membrane and is stripped from the surface. This fundamentally enhances sampling efficiency and eliminates the effect of surface/deposit material and impurities on the surface. In addition, the volume of the solution applied to a surface can be adjusted according to the sampling area, which avoids a decrease in sampling efficiency as the sampling area increases (e.g., which tends to occur when using swabs).

Water, ethanol and glycerol were used in the preparation of the PVA solution. These ingredients are chemically stable and exhibit no effect on most biological targets, including DNA molecules [48]. PVA has been widely used in the medical and pharmaceutical industries (e.g., in the preparation of eye drops and drugs) due to its low toxicity [43,49]. Glycerol has been widely used in cosmetics [50]. In the solutions proposed here, glycerol significantly increases the elasticity of the membrane.

### 4.3. Effect of the PVA Solutions on Microbial Viability

The sampling efficiencies exceeded 96% in all the measuring conditions (see Figure 4). Nevertheless, the 1 mL volume of PVA solution used during our laboratory sampling required 1–2 h for solidification. Surface microbes may multiply or degrade during this time.

Ethanol has biocidal efficacy against microbes. In contrast, short DNA fragments in surface sediments are relatively stable for hours [37,51] and can be precipitated in the presence of ethanol. Therefore, using the PVA solution (*m_e_* = 32%) to sample surfaces will not change the DNA concentration of the samples.

If the sampling target is living bacteria or fungi, a high mass ratio of ethanol in the PVA solution has antimicrobial effects on the living cells, whereas a solution with a very low mass ratio of ethanol may provide an aquatic environment for cell multiplication [52]. In this study, the optimal mass ratio of ethanol in the PVA solution for sampling surface *S. aureus* was determined. This ratio balanced the effects of bacterial multiplication and bacterial degradation; however, this mass ratio is only valid under the specific experimental conditions. In future studies, shortening the sampling time may simultaneously reduce the influence of the PVA solution on microbial activity, and a solution with a higher mass ratio of ethanol would accordingly be required. Thus, the optimal mass ratio of ethanol in the PVA solution should be adjusted according to the specific sampling conditions.

### 4.4. Limitations and Improvements

The proposed PVA solution-based sampling approach has some limitations. First, it is not suitable for sampling from water-absorbing materials, such as clothing, paper, food, concrete, human skin and most porous surfaces. In the case of surfaces with rough texture, the solidified membrane may break when stripped off. Second, some time is required for the drying of the solution applied to the surfaces. Applying less solution per unit area can accelerate its drying, but the thinner membrane formed will break more easily when stripped off. Third, ethanol in the solution accelerates its solidification, but a high concentration of ethanol has an antimicrobial effect.

In practice, the PVA solution is slightly irritating to human skin. Repeated or long-term application to human skin may cause a mild skin rash. While impurities on surfaces would not affect the collection of target substances, they may inhibit the detection using either PCR or plate assay. In addition, the consumables in detection, such as PCR primers and nutrient broths, should be adjusted and optimized according to the sampling targets.

In this study, we focused on introducing the new sampling approach, whereas its effect on few surfaces and species was measured because the high sampling efficiency exhibited in the measurement was considered due to a different mechanism in the newly presented approach, which could scarcely be affected by the surface/species material. The optimum *m_e_* in sampling living microbes was only measured for *S. aureus*, although it may also be affected by sampling conditions or as the sampling approach is optimized. In addition, we did not attempt to shorten the drying time because this was outside the scope of this study.

In future studies, we will develop techniques for accelerating the solidification of the PVA solution, such as mildly heating the surface, increasing the concentration of the solution or sampling under a negative pressure or an air flow. The ingredients in the solutions may be adjusted to fit specific requirements. For example, acetone, which has a low boiling point (BP = 56.5 °C), can be used as an additive to or a substitute for ethanol (BP = 78.4 °C) and water (BP = 100 °C) in PVA solutions to accelerate its solidification; propylene glycol can be used as a substitute for glycerol; and surfactants (e.g., fatty acids) may be added to efficiently sample lipophilic substances. Polyvinyl acetate can also be used to completely or partially replace the solute, as the former has shown a higher adhesiveness [53] and might improve sampling efficiency on surfaces with complex topographies.

In this study, a swab was used as an auxiliary tool to mix the sampling target with the PVA solution and to strip off the solidified membrane. Other tools can also be used as a substitute for swabs to detach the membrane. According to our experiment, no significant differences in sampling efficiency were found between using swabs and forceps to strip off the membrane.

## 5. Conclusions

This study proposed a new approach for sampling on non-absorbent surfaces. To sample a surface, a PVA solution was applied to the surface. The solution engulfed target substances as it solidified to a “soluble tape”, and the “tape” releases the substances when it is re-dissolved in water. As a complement of existing surface sampling methods, the new approach exhibited high sampling efficiency and consistency. The measured efficiencies exceeded 96% in collecting microspheres and DNA of different microorganisms from glass and stainless steel surfaces.

## Figures and Tables

**Table 2:** Experiment design in 15 sets of experiments.

No.	Aim	Sampling Target	Surface	Sampling Tools × Times	Quantification	Replicates
1	To measure *E* under different conditions	*S. aureus* DNA	Glass	PVA solution (*m_e_* = 32%) × 5 + Swab × 1	qPCR	5
2		*E. coli* DNA	Glass	PVA solution (*m_e_* = 32%) × 5 + Swab × 1	qPCR	5
3		Phage P22 DNA	Glass	PVA solution (*m_e_* = 32%) × 5 + Swab × 1	qPCR	5
4		*S. cerevisiae* DNA	Glass	PVA solution (*m_e_* = 32%) × 5 + Swab × 1	qPCR	5
5		*S. aureus* DNA	Stainless steel	PVA solution (*m_e_* = 32%) × 5 + Swab × 1	qPCR	5
6		*S. aureus* DNA	Glass	PVA solution (*m_e_* = 0) × 5 + Swab × 1	qPCR	5
7		*S. aureus* DNA	Glass	Swab × 6	qPCR	5
8		Microspheres	Glass	PVA solution (*m_e_* = 32%) × 6	Microscopy	5
9	To measure *S. aureus* viability in the PVA solutions	*S. aureus*	Glass	PVA solution (ethanol-free) × 1	Plate count	6
10		*S. aureus*	Glass	PVA solution (*m_e_* = 3%) × 1	Plate count	6
11		*S. aureus*	Glass	PVA solution (*m_e_* = 6%) × 1	Plate count	6
12		*S. aureus*	Glass	PVA solution (*m_e_* = 10%) × 1	Plate count	6
13		*S. aureus*	Glass	PVA solution (*m_e_* = 18%) × 1	Plate count	6
14		*S. aureus*	Glass	PVA solution (*m_e_* = 25%) × 1	Plate count	6
15		*S. aureus*	Glass	PVA solution (*m_e_* = 32%) × 1	Plate count	6

## Data Availability

Not applicable.

## Supplementary Materials

The following supporting information can be downloaded at: https://www.mdpi.com/article/10.3390/ijerph191912571/s1, Video S1: Video demonstrating the new sampling approach; Supplementary File S2: The amplification efficiencies in qPCR and the deviation in measuring sampling efficiency. Ref [54] is cited in the Supplementary file.
