# Supplementary material for: An Advanced Tape-Stripping Approach for High-Efficiency Sampling on Non-Absorbent Surfaces
Source: Int J Environ Res Public Health. 2022 Oct 1;19(19):12571. doi: 10.3390/ijerph191912571 (PMC9564726; doi:10.3390/ijerph191912571)
Supplement: Supplementary file 1 [file ijerph-19-12571-s001.zip › Supplementary File S2.pdf]

Supplementary File S2

An advanced tape-stripping approach for high-efficiency sampling on non-absorbent surfaces

Pengcheng Zhao [1], Pak-To Chan [1], Nan Zhang [1, 2], Yuguo Li [1, 3, *]

[1] Department of Mechanical Engineering, The University of Hong Kong, Hong Kong SAR, China

[2] Beijing Key Laboratory of Green Built Environment and Energy Efficient Technology, Beijing University of Technology, Beijing 100124, China

[3] School of Public Health, The University of Hong Kong, Hong Kong SAR, China

[*] Correspondence: liyg@hku.hk

I. Calibration of the qPCR amplification efficiency

For each pair of primers, the PCR efficiency was calibrated (Figure S1). During PCR, target DNA fragment is replicated in each heating cycle. The PCR efficiency was calibrated following the MIQE Guidelines [1], but the initial templet was 4-fold diluted. Thus, the PCR amplification efficiency (e) in Figure S1 is calculated as $e = 4^{-1/\mathrm{slope}} - 1$, in which the 'slope' represents the absolute value of the slope of the fitting line in Figure S1, where linear regression was performed, and the slopes in the plots are (a) –2.071; (b) –2.034; and (c) –2.302. Then, the e values are obtained as (a) 95.3%; (b) 97.7%; and (c) 82.6%, respectively.

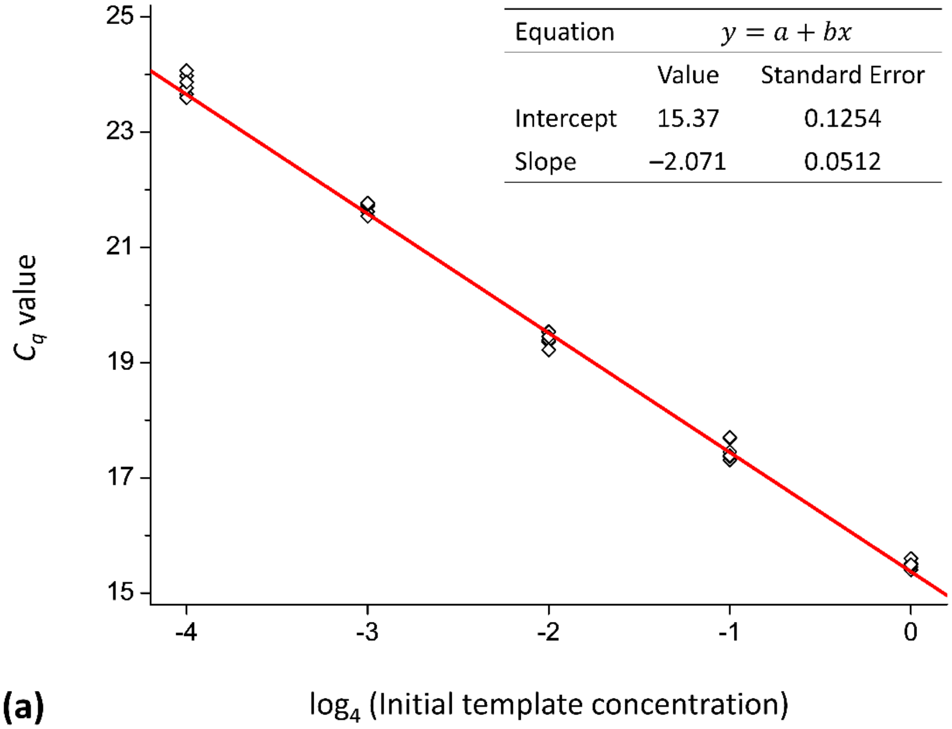

(a)

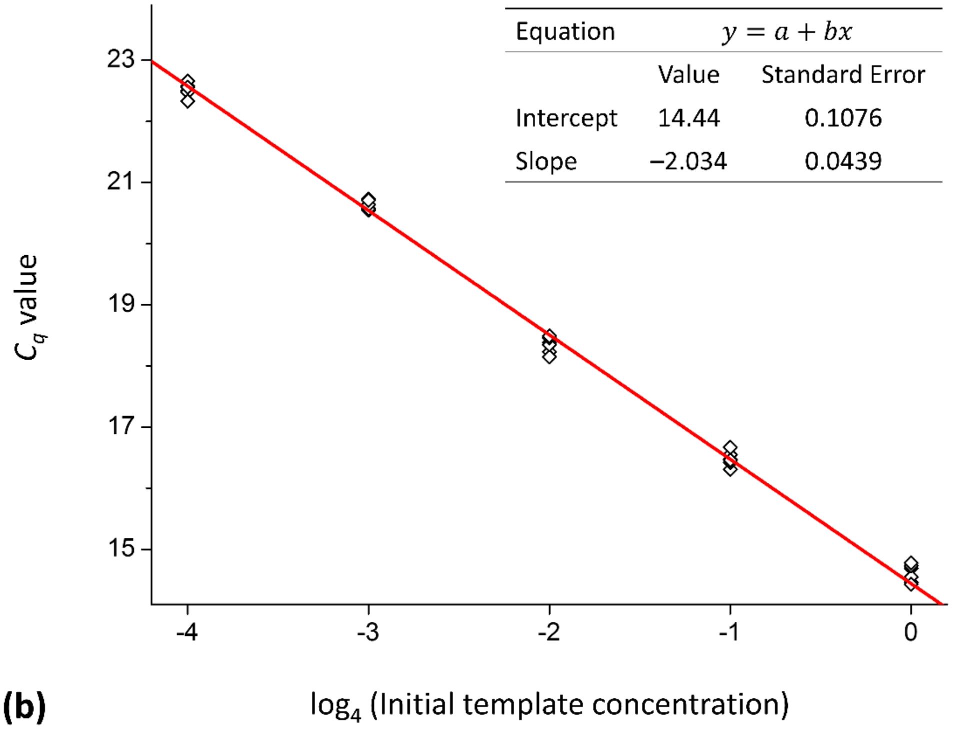

(b)

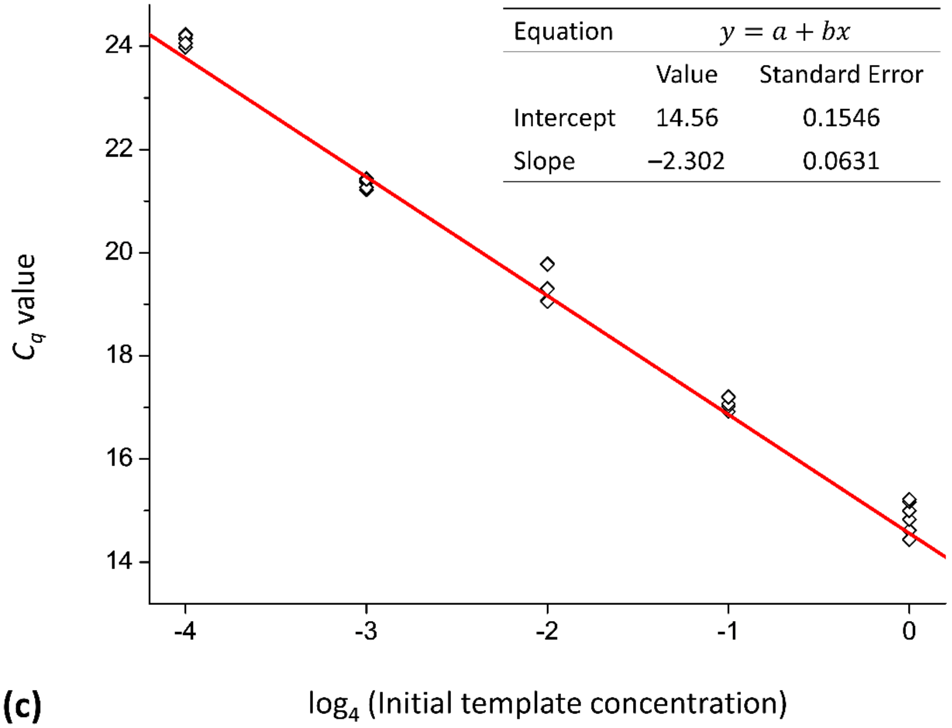

(c)

Figure S1 Calibration of PCR amplification efficiency for different pairs of primers.

(a) 341F and 518R. The universal primers in 16s rDNA used for prokaryote, used on *S. aureus* and *E. coli*;

(b) 14567F and 14617R. The primers used on phage P22; and

(c) ITS3-F and ITS4-R. The universal primers in 5.8s and 28s rDNA used for eukaryote, used on *S. cerevisiae*.

II. Deviation in the measurement of the sampling efficiency caused by bacterial multiplication/degradation

Assume the sampling target substances on a surface multiply(or degrade) with a constant speed, e.g., **d**C/**d**$t = \lambda C$, the quantity at the moment of detaching the PVA membrane (C) can be calculated using $C = e^{\lambda t}C_0$, in which C is varied by time (t), and the substances multiply (degrade) when $\lambda > 0$ (< 0). Assume a time of sampling spends t_0, the C can be obtained by Equation S1.

$$C = e^{\lambda t_0} C_0 \tag{A1}$$

where, t_0 is the time length for each sampling; and C_0 is the bacterial amount initially inoculated to the substrate surface.

The multiplication rate of the bacterial strain (R) during a sampling is defined in Equation S2. This is also mentioned in Section 2.6.2 in the main text.

$$R = \frac{C}{C_0} = e^{\lambda t_0} \tag{S2}$$

If we consider the multiplication rate (R) in calculating the sampling efficiency using Equation 1 in the main text, the microbes in each of the N-times sequential sampling ($\Delta C_1, \Delta C_2, \ldots, \Delta C_N$) as shown in Equations 1, 2 and 4 in the main text, are revised to Equations S3 (a–c).

$$\begin{cases} \Delta C_1 = E \times RC_0 \\ \Delta C_2 = E \times R(RC_0 - \Delta C_1) = ER[(1-E)R]C_0 \\ \Delta C_3 = E \times R[R(RC_0 - \Delta C_1) - \Delta C_2] = ER[(1-E)R]^2 C_0 \\ \quad \vdots \\ \Delta C_n = ER[(1-E)R]^{n-1} C_0 \end{cases} \tag{A3, a}$$

$$\Rightarrow \quad \sum_{i=1}^{n} \Delta C_i = ERC_0 \frac{1 - [(1-E)R]^n}{1 - (1-E)R} \tag{A3, b}$$

$$\Rightarrow \quad \frac{\Delta C_n}{\sum_{i=1}^{N}(\Delta C_i)} = \frac{[1-(1-E)R] \times [(1-E)R]^{n-1}}{1 - [(1-E)R]^N} \tag{A3, c}$$

Set $\boldsymbol{E' = 1 - (1 - E) \times R}$, as shown in Equation S4.

$$E = 1 - (1 - E')R^{-1}$$

$$\Rightarrow \quad \begin{cases} E' - E = (1-E) \times (1-R) \\ E - E' = (1-E') \times (1-R^{-1}) \end{cases} \tag{A4}$$

Then, Equation S3 (c) can be simplified to Equation S5, which has the same structure with

Equation 4 in the main text.

$$\frac{\Delta C_n}{\sum_{i=1}^{N}(\Delta C_i)} = \frac{E'(1-E')^{n-1}}{1-(1-E')^N} \tag{A5}$$

In Equation S5, *E*' can be considered as a **calculated sampling efficiency** with the occurrence of bacteria multiplication (or degradation). Combining Equations S4 and S5, the deviation in evaluating the **sampling efficiency *E*** is summarized as follows

- if $R = 1$, it indicates a stable amount of the bacterial strain. Then, $E' = E$, meaning that the sampling efficiency would be estimated accurately;
- if $R > 1$, it indicates a multiplication of the bacterial strain. Then, $E' < E$, meaning that the sampling efficiency would be underestimated; and
- if $R < 1$, it indicates a degradation of the bacterial strain. Then, $E' > E$, meaning that the sampling efficiency would be overestimated.

References

1. Bustin, S. A.; Benes, V.; Garson, J. A.; Hellemans, J.; Huggett, J.; Kubista, M.; Mueller, R.; Nolan, T.; Pfaffl, M. W.; Shipley, G. L.; Vandesompele, J.; Wittwer, C. T., The MIQE guidelines: minimum information for publication of quantitative real-time PCR experiments. *Clin. Chem.* **2009,** 55, (4), 611-622.
